# Assessing Older Adults’ Daily Mobility: A Comparison of GPS-Derived and Self-Reported Mobility Indicators

**DOI:** 10.3390/s19204551

**Published:** 2019-10-19

**Authors:** Michelle Pasquale Fillekes, Eun-Kyeong Kim, Rieke Trumpf, Wiebren Zijlstra, Eleftheria Giannouli, Robert Weibel

**Affiliations:** 1 Department of Geography, University of Zurich, Winterthurerstrasse 190, 8057 Zurich, Switzerland; eun-kyeong.kim@geo.uzh.ch; 2 University Research Priority Program “Dynamics of Healthy Aging”, University of Zurich, Andreasstrasse 15, 8050 Zurich, Switzerland; 3 Institute of Movement and Sport Gerontology, German Sport University Cologne, Am Sportpark Müngersdorf 6, 50933 Cologne, Germany; r.trumpf@dshs-koeln.de (R.T.); zijlstra@dshs-koeln.de (W.Z.); e.giannouli@dshs-koeln.de (E.G.); 4 Department of Geriatric Psychiatry and Psychotherapy, LVR Hospital Cologne, Wilhelm-Griesinger-Straße 23, 51109 Cologne, Germany

**Keywords:** healthy aging, mobility, mobility indicators, real-life, convergent validity, global positioning system, self-reports, sensor-based, time out of home, activity location

## Abstract

Interest in global positioning system (GPS)-based mobility assessment for health and aging research is growing, and with it the demand for validated GPS-based mobility indicators. Time out of home (TOH) and number of activity locations (#ALs) are two indicators that are often derived from GPS data, despite lacking consensus regarding thresholds to be used to extract those as well as limited knowledge about their validity. Using 7 days of GPS and diary data of 35 older adults, we make the following three main contributions. First, we perform a sensitivity analysis to investigate how using spatial and temporal thresholds to compute TOH and #ALs affects the agreement between self-reported and GPS-based indicators. Second, we show how daily self-reported and GPS-derived mobility indicators are compared. Third, we explore whether the type and duration of self-reported activity events are related to the degree of correspondence between reported and GPS event. Highest indicator agreement was found for temporal interpolation (*Tmax*) of up to 5 h for both indicators, a radius (*Dmax*) to delineate home between 100 and 200 m for TOH, and for #ALs a spatial extent (*Dmax*) between 125 and 200 m, and temporal extent (*Tmin*) between 5 and 6 min to define an activity location. High agreement between self-reported and GPS-based indicators is obtained for TOH and moderate agreement for #ALs. While reported event type and duration impact on whether a reported event has a matching GPS event, indoor and outdoor events are detected at equal proportions. This work will help future studies to choose optimal threshold settings and will provide knowledge about the validity of mobility indicators.

## 1. Introduction

Because of the rapidly aging societies around the world, promoting healthy aging has become an ever-important research endeavor [1]. Being able to participate in social interactions, as well as maintaining an active and independent lifestyle are central components of healthy aging [2,3,4,5]. These components essentially rely on the individuals’ capacity of remaining mobile out of home [6]. Thus, assessing older adults’ daily mobility, defined for this paper as the everyday spatiotemporal patterns of an individual’s movement in their environment, enables insight into an individual’s level of participation in natural, built, cultural, and social environments [3,7,8]. One common way to assess daily mobility, is by using mobility indicators that quantify different aspects of an individual’s mobility [9,10]. Time out of home (TOH) and number of activity locations (#ALs) are two such indicators which have been widely applied in the field of health- and aging-related sciences as they have been found to be associated with older adults’ cognitive, physical, and emotional functioning [11,12,13,14,15,16]. TOH is an indicator assessing how much time an individual spends outside their residence, whereas activity locations (ALs) refer to the places that an individual has visited to perform an activity (e.g., waiting for a train, visiting a friend, shopping) [17].

TOH and #ALs can be measured and assessed by various instruments. Commonly used tools are self-reports, e.g., questionnaires and diary-based surveys, and location sensors, e.g., the global positioning system (GPS). Each of those instruments has different strengths and weaknesses. While self-reports can capture semantic attributes of activities beyond physical properties (i.e., location and time) but entail cognitive biases (e.g., recall bias), GPS collects precise location and time and is free from such human biases. However, GPS is subject to potential device failure (e.g., signal loss) or human mistakes (e.g., missing device), and requires data processing algorithms for extracting semantically plausible activity information. For instance, depending on the thresholds of an automatic activity detection algorithm—e.g., minimum time duration (*Tmin*), maximum distance (*Dmax*), and maximum gap duration (*Tmax*) of GPS fixes—the spatio-temporal extent of detected activities may vary. Consequently, different assessment instruments often yield different data and, further, inconsistent results of mobility indicators including TOH and #ALs.

This raises the important question that which indicators (e.g., TOH, #ALs) using which assessment tools (e.g., self-reports, GPS) do represent more valid representations of older adults’ mobility? To evaluate whether indicators portray the construct that one intends to quantify, measures of indicator agreement (e.g., Bland Altman statistics, correlation) inform about whether indicators assessed with different tools, but designed for the same construct, yield similar results. Comparison studies on self-reported and GPS-derived #ALs are still limited (i.e., focused on spatial correspondence [18,19], or aggregate measures [16]), and no study was identified comparing self-reported and GPS-based TOH. Therefore, it is imperative to compare TOH and #ALs derived from self-reports and GPS. In doing so, impacts of algorithm threshold settings should be considered [20,21]. While thresholds can drastically change the results of GPS-based TOH and #ALs, many studies have ignored the effect of the thresholds [12,22,23].

This study’s main aim is to evaluate how self-reported and GPS-based daily mobility indicators compare in a sample of older adults, without making any a priori assumptions about the accuracy of either of the data sources. To this end, we extract the two daily mobility indicators TOH and #ALs from one-week of diary and GPS data of 35 older adults. More specifically, our work aims to make the following contributions:

1. We investigate in a sensitivity analysis how the choice of thresholds (*Tmin*, *Dmax*, *Tmax*) affects the indicator agreement between self-reported and GPS-based indicators.
2. We explore the convergent validity for both mobility indicators by comparing the self-reported and GPS-derived daily mobility indicators.
3. We explore whether self-reported activity characteristics (i.e., the duration or type of an activity) affect a self-reported event, finds a corresponding, temporally overlapping (i.e., matching) GPS event.

## 2. Self-Reports and GPS Sensors for Assessing Daily Mobility

### 2.1. Self-Reports

Various instruments exist to assess individuals’ daily mobility and depending on the study goal one or the other might be more suitable. Most of these methods today rely on self-reports, including questionnaires and diary-based surveys. Questionnaires typically assess habitual mobility in a single occasion and in a retrospective manner via mobility questionnaires (e.g., life-space assessment (LSA) [24]) or interactive map-based questionnaires (e.g., VERITAS [25]). These retrospective questionnaires are prone to recall bias and unable of capturing the day-to-day variation and complex nature of mobility behavior [21,26,27]. Daily travel diaries are designed for a participant to fill out continuously throughout the day or at the end of each study day to report information on location, time, and attributes of each of an individual’s daily activities (Table 1). Such frequently reported information allows examining shorter-term fluctuations in an individual’s mobility patterns [28]. Semantic information reported on each activity and trip (e.g., activity type, present persons, transport mode) enables semantically enriched analysis. However, diary-based surveys expose a range of disadvantages: participant burden because of the time-consuming reporting process [29], failing to report activities correctly because of subjective bias, lack of care, recall bias, or social desirability bias [30,31,32], and missing exact locational data (i.e., geographic coordinates) [18,25,29]. Self-reported assessments over long periods are theoretically possible, however, in practical terms they are feasible only to a limited degree, as self-reports require continuous high participant involvement.

### 2.2. GPS as a Location Sensor

Wearable global positioning system (GPS) technology is increasingly used to objectively assess individuals’ visited locations and travels undertaken [11,15,33,34]. GPS sensors continuously collect location fixes—each represented by a position coordinate together with a timestamp (date and time). GPS tracking enables the observation of daily mobility over potentially long time periods, with high spatio-temporal resolution and minimal participant involvement. Despite its benefits, GPS tracking faces several challenges. Low participant compliance, battery life issues, and interrupted satellite signals may lead to data gaps and, consequently, to high attrition rates [9,35]. As no semantic information is collected by GPS, extracting meaningful information is necessary through multi-step data processing including aggregations of GPS data points regarding space, time, and behaviors, which is time-consuming and requires technical skills [20]. Unfortunately, there are no standardized GPS data processing procedures including threshold setting for automatic detection of activity locations and trips [9,36].

### 2.3. GPS Data Processing and Threshold Setting

**Figure 1:**
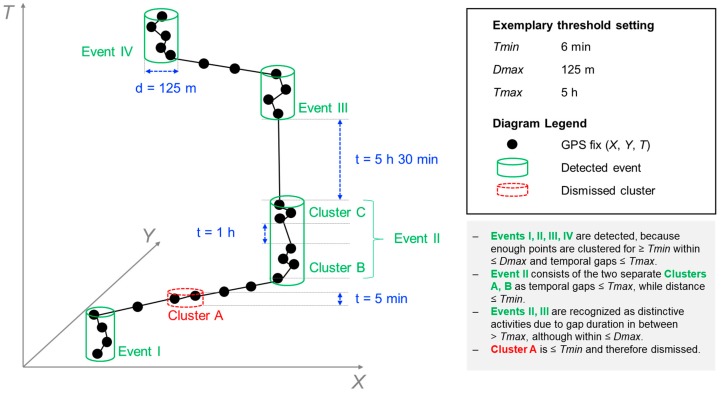
Schematic representation of automatic activity location detection based on spatial (*Dmax*) and temporal parameters (*Tmin*, *Tmax*), depicted in a space–time cube. The example thresholds used in this illustration (*Tmin* = 6 min, *Dmax* = 125 m, *Tmax* = 5 h) are the ones that were derived from the sensitivity analysis (see Section 4.2).

GPS data processing is the key to accurate assessment of GPS-derived daily mobility. TOH and #ALs, the two mobility indicators in the focus of this study, are computed based on spatio-temporal extent of home and activity locations. These features are usually automatically detected from GPS fixes. For TOH, GPS fixes outside a circular buffer around home should be identified in consideration of GPS error and then the time between out-of-home GPS fixes is summed up. For #ALs, activity locations should be inferred and more complex algorithms are required [17,37,38,39,40]. Figure 1 schematically shows how a typical algorithm to detect activity locations works. For both indicators, the following spatial and temporal thresholds used as input parameters of a processing algorithm play a significant role in such automatic detection [17,38,41,42]:

- Minimum time duration (*Tmin*): *Tmin* defines the minimum time spent out of home or in an activity location in order to count as valid TOH or AL event, respectively.
- Maximum distance (*Dmax*): For TOH, *Dmax* is a radius defining a buffer around home. All fixes within the buffer count as home, all fixes outside of the buffer count as out of home (OH). For #ALs, *Dmax* defines the maximum spatial region within which the individual can move (respectively the GPS signal is allowed to wander) in order to be defined as an AL.
- Maximum gap duration (*Tmax*): For both indicators, *Tmax* defines the maximum duration of a gap in GPS data over which interpolation is invoked if the remaining conditions are met (i.e., *Tmin*, *Dmax*).

Although this does not need to be the case, we expect that thresholds to accurately detect TOH and AL events are both falling in a similar range, as from a purely spatio-temporal perspective they are both defined as a stay with a minimum duration within a spatial region defined by a maximum radius. In the literature, thresholds applied vary substantially between studies and are often presented without justification. For *Tmin* values typically range between 3 and 30 min and for *Dmax* between 25 and 500 m [17,43,44,45,46,47,48]. *Tmax* is often not dealt with or explicitly mentioned, although temporal gaps in GPS data are very frequent as individuals typically spend a lot of time in GPS-denied areas (e.g., indoors) [45]. Interpolation in GPS data is often feasible with little risk of introducing errors [43]. Whenever interpolation is introduced, 1 h seems to be the maximum temporal gap for which interpolation is done [2,19,45]. While in the referenced literature, interpolation was done by filling in gaps with synthetic data points on the level of the raw GPS trajectory, this paper takes an approach of filling in data gaps on the level of detected mobility events. That is, interpolation (or gap filling) takes place in a temporal fashion, merging two subsequent events separated by a temporal gap, if the gap is smaller than *Tmax* and if *Tmin* and *Dmax* are met at the same time.

### 2.4. Self-Reported versus GPS-Derived Mobility Indicators

While it is commonly accepted knowledge that GPS outperforms self-reports, e.g., in terms of assessing the exact geometry of mobility whereas self-reports outperform GPS, e.g., in assessing additional mobility-related semantics (e.g., type of activity), there is no agreement in the literature regarding which method performs better in assessing aggregate mobility indicators such as TOH or #ALs. To compare indicators, construct validity is often evaluated regarding whether an indicator represents the construct that one intends to measure. One way of assessing the construct validity is to examine the so-called convergent validity. It examines whether indicators aiming at assessing the same construct, but using different instruments (e.g., self-reports versus GPS) yield similar results [18,49,50]. Research comparing self-reports to GPS is especially to be found in the field of transportation research where travel durations using different transport modes are compared. A systematic review found that these tend to be larger when self-reported rather than derived from GPS data [51].

In the context of health- and aging-related research, Fillekes et al. [52] also found self-reported tools to exceed GPS-based travel duration, while daily self-reported life space (assessing maximum distance traveled from home) fell slightly below its GPS-derived counterpart. Boissy [20] found weak to moderate correlations (r = 0.3–0.4) between LSA scores and various GPS-derived life-space indicators (including standard deviational ellipse area, distance covered in vehicle). Wolf et al. [16] found moderate correlations (0.4) when comparing #ALs derived from GPS and diaries aggregated over the entire study period of 4 days. Shareck et al. [18] found high levels of agreement in terms of spatial correspondence of ALs reported in a questionnaire on regularly visited locations and one week of GPS data. Similarly, Kestens et al. [19] found good spatial correspondence between self-reported regularly visited ALs with a map-based tool and one week of GPS data. A recent study comparing self-reported to GPS-derived time spent outdoors (i.e., outside of any building), found self-reports to massively exceed GPS-derived measures based on indicators aggregated over the entire study period per participant [53].

In summary, only few comparison studies have been carried out in the health and aging domain, and most of these have focused on “global” mobility indicators that characterize aggregate mobility behaviors over the entire study period [9]. These days, however, research interest turns increasingly to mobility indicators measured over shorter intervals (e.g., day to day) in order to observe within-person fluctuations of mobility [22]. Such indicators are potentially derivable from data with high spatio-temporal granularity as obtained via positioning sensors or real-life assessments. Hence, there is a need for comparison studies for mobility indicators using shorter intervals. Knowledge regarding the degree of agreement between self-reported and GPS-derived mobility indicators will help to improve the understanding of the reliability of such indicators. This, in return, allows to determine with which level of confidence such mobility indicators can be interpreted in their own right and how reliably they can be related to other healthy-aging-relevant outcomes. Moreover, such knowledge contributes to determining to which degree these two methods should be used in complementary fashion, respectively, to which degree they could potentially substitute each other.

## 3. Methods

### 3.1. Study Design and Participant Recruitment

Data collection was part of the “ChronoSense Study,” a cross-sectional study, aiming to investigate circadian rhythms of mobility patterns in community-dwelling older adults using wearable sensors. The study was designed in compliance with the principles expressed in the Declaration of Helsinki and has been approved by the Ethics Committee of the German Sport University Cologne (registration number 156/2017). All participants provided written informed consent. To recruit study participants, information on the project was emailed to local senior citizens’ networks and presented in senior’s sport groups. Furthermore, persons who expressed interest in participating in studies of the Institute of Movement and Sport Gerontology in the past were invited by email or telephone call. To be eligible for the study, subjects had to be community-dwelling, aged 65 years or older, and subjectively healthy. Persons with any acute or severe mobility impairment, cardiovascular disorder, cognitive disorder or neurological disease (based on self-report) which could interfere with functional mobility were excluded from participation. Furthermore, persons with a score of 24 or fewer points in the mini-mental state examination (MMSE) [54] were excluded from the study [55]. In total, 38 eligible persons participated but only 35 persons were included in the study after exclusion of three early dropout participants.

Of the 35 participants 17 were women and 18 were men. Participants’ mean age was 70.6, ranging from 65 to 83 years. Participants indicated as their highest level of education: primary school (7; 20%), secondary school (6; 14%), university of applied sciences (6; 14%), and university (16; 46%). Body mass index (BMI) was 26.92 +/− 4.9, and the majority of the participants (29 out of 35) suffered from at least one chronic disease.

### 3.2. Data Collection

Data collection had three stages of pre-registration, one-week registration, and post-registration. In the pre-registration, each participant visited the laboratory to take a baseline questionnaire on her/his socio-demographics and home address and receive mobility diaries for self-reports and smartphone (SP) for GPS data collection. Then, actual self-reporting and GPS-tracking were conducted for a week. Afterward, the participants revisited the laboratory to return the diaries and smartphone for data download.

Different instructions were given for self-reporting and GPS-tracking. The detailed execution for each approach is as follows. For self-reports, each participant was given two separate paper diaries for TOH and ALs and asked to fill them out multiple times or at the end of each study day. In the TOH diary, each participant was asked to report the times when they left home for an activity and came back (i.e., the start and end times of each TOH event) as well as whether they carried the SP during each of the reported TOH events. In the AL diary, each participant was requested to report start and end times and the names of all visited places where they stayed beyond 3 min, but in an explicitly stationary state, including long transient stops at a train/tram station, sitting on a park bench, and shopping in a supermarket. A minimum of 3 min for an activity location was chosen in order to exclude extremely short stops, such as waiting at a traffic light. All types of traveling (e.g., walking in a park) were to be excluded in the reports.

GPS data were recorded with a sampling frequency of 1 s using the application (app) GPSLogger, version 89 [56] on a Samsung Galaxy SIII GT-9301. The app was set to start automatically whenever the SP was switched on. Each participant was asked to turn on the SP in the morning after waking up and to turn it off in the evening before going to bed. They were instructed to carry the SP with them, whenever they were out of their homes. In order to achieve the longest possible battery life, the participants were instructed to charge the SP when they were at home. All participants performed a 15-min familiarization session which covered how to switch the SP on and off, charge it, use the touch screen, and to make sure the app was running. Additionally, each participant received a project-specific user manual and was offered the opportunity to contact the instructors in case they had questions or faced complications regarding smartphone use.

### 3.3. Self-Reported Mobility Indicators

Self-reports were processed through the following procedures to measure mobility indicators. In a first step, TOH events out of the study period and of duration ≤3 min were deleted. AL events were excluded if at least one of the following conditions was met: out of study period, missing start/end time, duration ≤3 min, non-stationary activities/movements, activities that took place at home, and false reports (i.e., entries not related to mobility). Moreover, an activity category was assigned to each AL based on participants’ annotations, using an adapted activity location classification from Kaspar et al. [22]. This classification was specifically developed for a study sample of older adults and consists of the following categories: work, commercial, social, personal care, culture/religion/education, recreation, transportation, unassignable. The duration of the valid TOH events were summed to an indicator reflecting total reported daily duration spent out of home (“REP TOH”). The valid activity locations per day were counted per day to represent the self-reported indicator daily number of activity locations (“REP #ALs”). Study days were segmented at midnight.

### 3.4. GPS-Based Mobility Indicators

GPS data processing consisted of the following seven steps: removal of outliers, splitting into daily trajectories, exclusion of invalid days, validation of geocoded home location, extraction of TOH events, extraction of ALs, and aggregation to the daily indicators “GPS TOH” and “GPS #ALs”.

**Outlier removal:** We removed outliers from the individual GPS fixes featuring speeds above 330 km/h. This corresponds to the speed limit of high-speed trains in Germany.

**Day segmentation:** The weekly GPS trajectories were split into daily segments at midnight to correspond to self-reported indicators.

**Exclusion of invalid days:** Same as in existing GPS-based studies [57,58,59], study days were excluded if the daily registration period, that is, the time between first and last GPS fixes of a day was less than 9 h. Moreover, further computation was only done for participants with at least 2 days of valid GPS data.

**Home validation:** The geocoded self-reported address was validated by comparing it to the GPS-derived home location. To extract the GPS-based home location, similar as in a previous publication [9], we used DBSCAN [60,61] with minimum number of points = 3 and epsilon distance = 60 m to extract clusters based on all first morning and last evening fixes of every valid day. If multiple clusters were identified, the cluster closest to the self-reported home location was chosen as the GPS-based home location. Finally, homes (and consequently participants) were only counted as valid, if the distance between the address-based and GPS-based home was smaller than 150 m. For further computation, we replaced the address-based home with the GPS-based home, in case the latter reflected more precisely the GPS fixes (more GPS fixes falling into a 60-m-buffer of the GPS-based than the address-based home).

**Figure 2:**
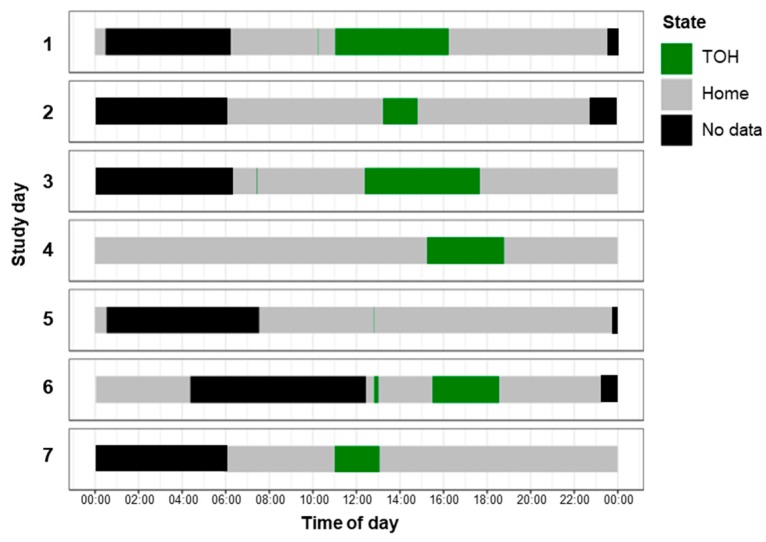
Temporal view of global positioning system (GPS) detected time out of home (TOH) and home events for 7 days of one participant (*Dmax* = 125 m, *Tmin* = 6 min, *Tmax* = 300 min).

**Extraction of TOH events:** As introduced above, we have identified TOH events using the following three parameters: *Dmax* defining the radius for the home buffer to distinguish between home and out-of-home (OH) fixes, *Tmin* defining the minimum TOH event duration to be considered valid, and *Tmax* defining the temporal gap for which we temporally interpolated. Temporal interpolation simply consisted in filling in the binary state (i.e., either “home” or “OH”) into data gaps of up to *Tmax*, in case the previous and subsequent fix had both been identified with the same state. Figure 2 shows the identified TOH events along the temporal axis across the seven study days of one participant based on the spatial and temporal thresholds obtained in the sensitivity analysis (see Section 3.5 and Section 4.2).

**Figure 3:**
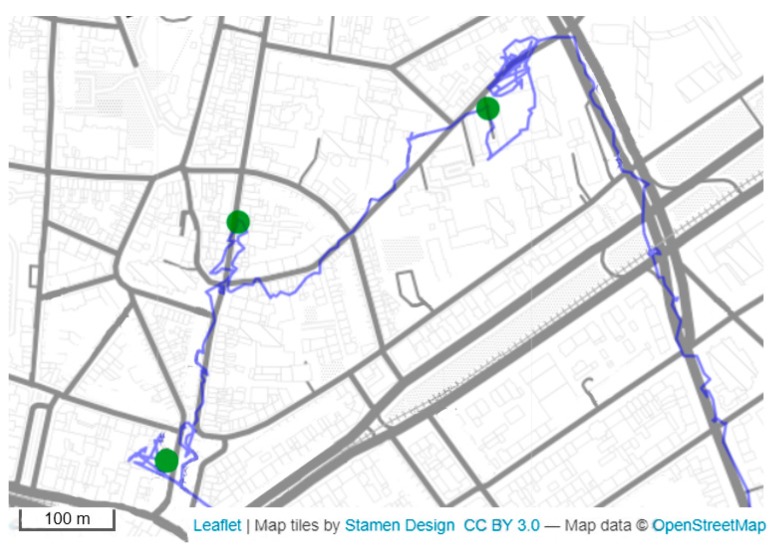
Some detected activity locations (green dots) for one study day of one user (*Dmax* = 125 m, *Tmin* = 6 min, *Tmax* = 300 min). This illustration also shows the typical positional data quality of the raw, unprocessed GPS data.

**Extraction of ALs:** To detect activity locations (Figure 3), we used the stop detection algorithm proposed by Montoliu et al. [38]. The algorithm’s adjustable parameters are intuitive and reflect those most often used in health and aging research [41,62,63,64]. Moreover, in contrast to many other algorithms, it is capable of dealing with temporal gaps in GPS data [65]. Montoliu’s algorithm is a time-based clustering algorithm using the three above-introduced input parameters: *Dmax* represents the maximum allowed distance of a GPS fix from the first GPS fix of a stop cluster; *Tmin* represents the minimum duration of a group of identified GPS fixes in order to count as a stop; and finally, *Tmax* represents the maximum allowed time gap between consecutive location points to be considered as a part of the same stop cluster (see Figure 1). A detected stop episode is represented by the position of the median latitude and median longitude of the included GPS fixes, and by the timestamps defining the start and end of the stop. In a subsequent step, episodes not fulfilling the stop criteria and <3 min were interpreted as “noise” (e.g., indoor signal wandering [66]). Three minutes is a commonly applied threshold for the minimum move duration [58,67]). Consecutive stops previously interrupted by such short “noise” episodes, were merged if spatial distance and temporal difference between those two stops were smaller than *Dmax* and *Tmax*, respectively. Finally, we used *Dmax* as a radius around an individual’s home location to exclude stops referring to home, as we were only interested in detecting out-of-home activity locations.

**Aggregation to daily indicators:** The duration of the TOH events was summed for each day to the GPS-derived indicator “GPS TOH” representing total daily duration spent out of home. The identified ALs were counted for each day to represent the GPS-derived indicator “daily number of activity locations” (“GPS #ALs”).

### 3.5. Sensitivity Analysis for Dmax, Tmin, Tmax

**Figure A1:**
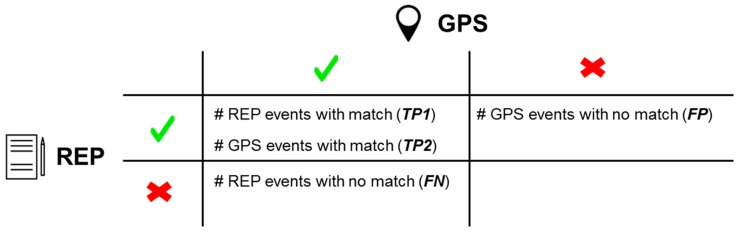
Confusion matrix for data with no ground truth. Note: REP = self-reported, GPS = GPS-based, TP = true positive, FN = false negative, FP = false positive, # = number.

**Figure A2:**
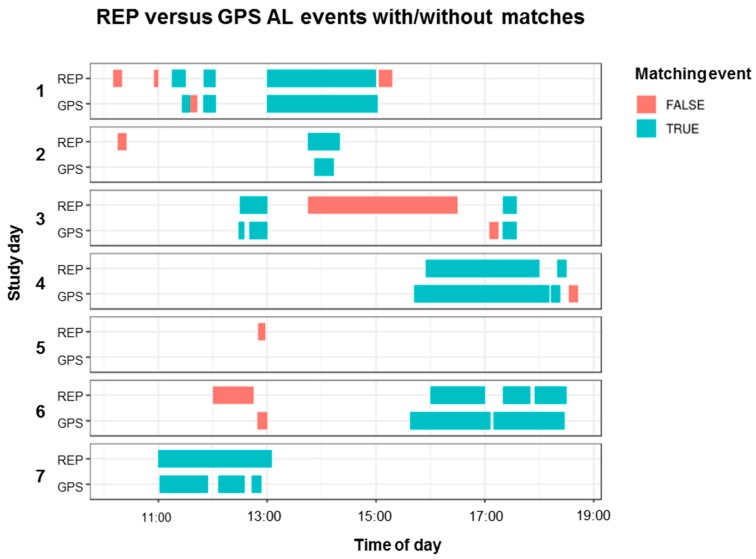
Identification of matched events exemplified by one week of self-reported (REP) and GPS-based AL events (same participant as in Figure 5 of main paper).

**Figure A3:**
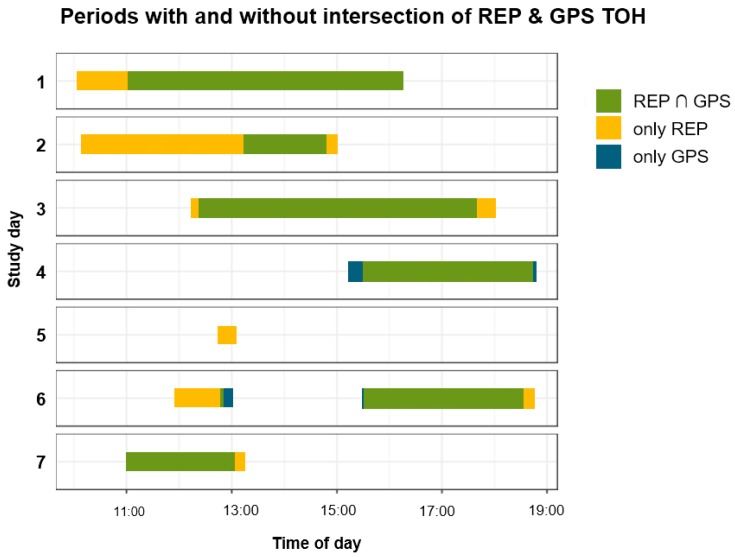
Identification of IoU based on one week of TOH data of one participant (same participant as in Figure 4 of main paper).

Since there is no commonly accepted standard for the thresholds used to extract TOH events and ALs from GPS, we performed a sensitivity analysis with the aim of evaluating how this impacts agreement between self-reported and GPS-derived TOH and AL events. To assess indicator agreement, we have used the measures F1 and IoU (Intersection over Union), also referred to as Jaccard coefficient. Both measures are computed at the event level and evaluate events as agreeing if they temporally overlap (i.e., match). F1 is a harmonic measure of precision and recall also used in Hwang et al. [66] (Figure A1 and Figure A2 in Appendix A). Recall was determined as the number of correctly reported (i.e., with GPS match) events out of the total number of reported events [41]. Precision was calculated as the number of correctly detected events (i.e., with reported match) out of the total number of events detected by GPS. Optimizing F1 means maximization of the number of events with a match (“true positives”), while minimizing the number of events that were only reported (“true negative”) or GPS-derived (“false positives”), respectively. While this is very sensible for the #ALs which is concerned with counts, for TOH—oriented toward duration—optimization of IoU is more relevant (Figure A3 in Appendix B). IoU maximizes intersection periods (overlapping GPS and reported events) while minimizing periods that were either only reported or only derived from GPS [17]. Both indicators range between 0.0 and 1.0, with 1.0 being the maximum, implying exact overlap between reported and GPS events in terms of counts or duration, respectively. Based on visual inspection of the two curves describing indicator agreement (F1 and IoU) as a function of the respective thresholds chosen, we selected the thresholds that optimized simultaneously both agreement measures for both mobility indicators. Because a stay at home or a stay in an activity location looks very similar in terms of spatio-temporal characteristics (i.e., not moving out of a restricted zone for at least a minimum amount of time), we expect that similar parameter settings will typically optimize agreement between self-reports and GPS for both mobility indicators.

**Figure A4:**
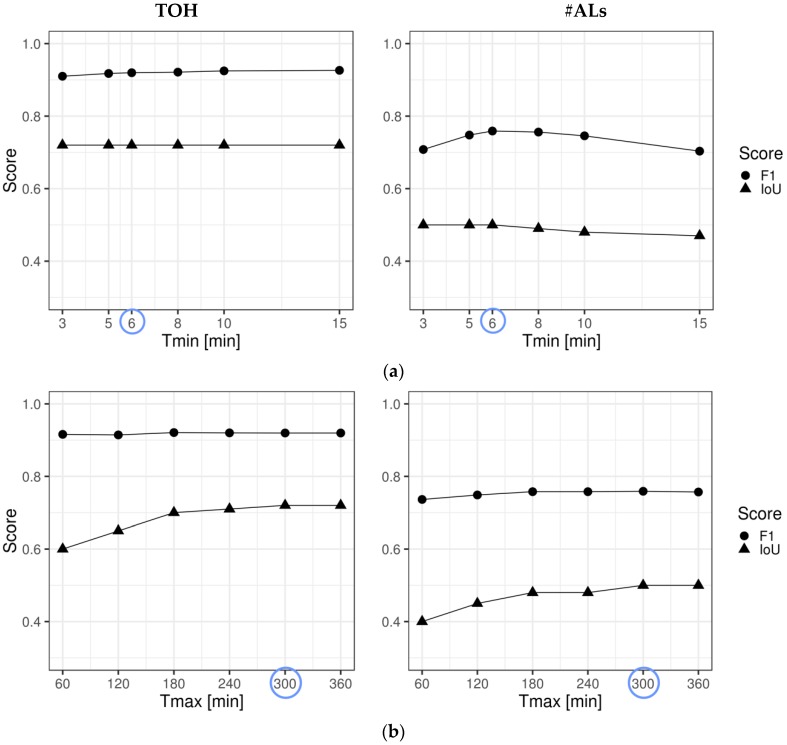
Confirmation of optimized thresholds by reverse stepwise iteration of *Tmin* and *Tmax*. (**a**) Confirmation of *Tmin* based on optimized *Tmax* = 300 min and *Dmax* = 125 m. (**b**) Confirmation of *Tmax* based on optimized *Dmax* = 125 m and *Tmin* = 6 min.

We started with an initial parameter setting and iteratively optimized the following parameters while holding the remaining two parameters constant (1) *Tmax*; (2) *Tmin*; (3) *Dmax*; (4) re-confirmation of *Tmin*; (5) re-confirmation of *Tmax*. The constant parameters were replaced after every step with the threshold that had been identified to optimize indicator agreement in the previous step. We chose the following initial threshold setting for the stepwise optimization (Section 4.2) and re-confirmation (see Appendix D, Figure A4) process:

- *Tmin* = 3 min: Corresponds to the minimum duration of an activity location instructed to participants and has often been used elsewhere [17,66,68,69,70];
- *Tmax* = 1 h: Typical maximum gap length found in the literature upon which GPS data is interpolated [9,19,66];
- *Dmax* = 100 m: Good indicator agreement was obtained for 100 m in Cich et al. [17] and Hwang et al. [66] testing multiple thresholds in their stop detection algorithms. Also 100 m was used in Harada et al. [11,71] to identify TOH.

### 3.6. Inclusion Criteria

Out of the initial 245 study days (35 participants × 7 days), we excluded days and participants based on the following criteria (see Section 4.1):

**Exclusion of GPS days**: GPS days were invalidated, if as aforementioned the registration period was <9 h or if participants indicated to not have worn the SP for at least one reported OH event of the respective day.

**Exclusion of reported (REP) days**: Study days of participants who did report events for less than two days overall, were deemed invalid for the respective indicator. Moreover, days with reports of TOH and AL that were contradictive were invalidated. That is, REP TOH days were considered invalid, if no TOH was reported while at least one AL was reported for the same day. Similarly, REP AL days were invalidated, if no AL was indicated while >30 min of TOH was reported.

**Included days/participants**: Eventually, we only ran comparison analyses for days of participants who had at least 2 days with matching valid REP and GPS indicators.

### 3.7. Statistical Analyses

**Figure 4:**
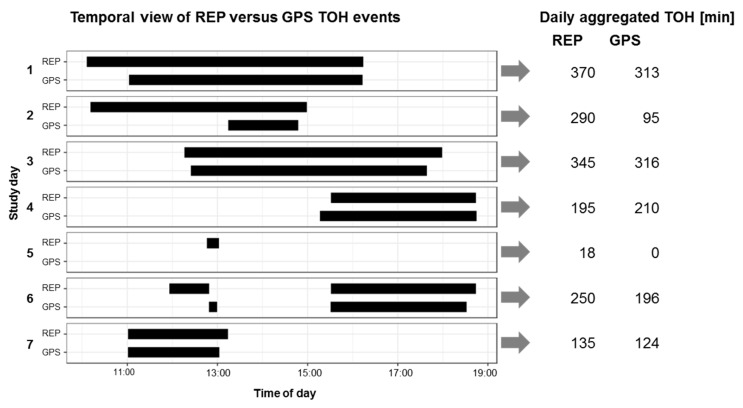
Daily temporal view of REP versus GPS TOH events (**left**) and daily aggregated indicators REP TOH versus GPS TOH (**right**) for one participant (one week).

**Figure 5:**
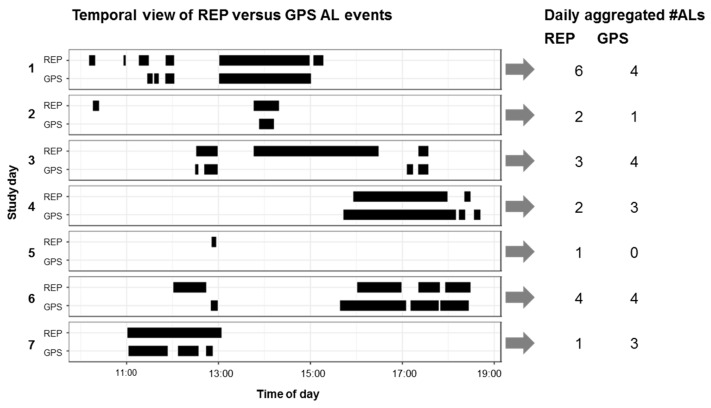
Daily temporal view of REP versus GPS activity locations (**left**) and daily aggregated indicator REP #ALs and GPS #ALs (**right**) for one participant (one week).

Exemplary resulting TOH events and ALs are shown for one participant in Figure 4 and Figure 5, respectively, once as detailed horizontal bars (left) and once aggregated to daily indicators in table format (right). In order to evaluate the convergent validity of daily self-reports versus GPS, besides the descriptive statistics of the differences between daily reported and GPS-based measures, the following measures of agreement were computed: Bland Altman statistics for multiple observations per individual [72]; Spearman correlations across all study days, and aggregated over individuals (iCorr) [52]; and interrater intraclass correlation coefficient (ICC). These measures are described in detail including conventions for interpretation of association strength in Table A1 in Appendix C. We do not make any assumption about any data source being the reference/ground truth.

While all of the abovementioned statistics are based on the aggregated daily mobility indicators, we computed further measures on the event level, in order to gain further insight regarding explanations for potential differences between REP and GPS indicators. Besides the aforementioned precision, recall, F1 and IoU measures, we investigated the proportion of duration as well as the counts of matching REP and GPS events. Eventually, we investigated whether certain characteristics of the reported events (duration, activity type, indoor versus outdoor) have an impact on whether a reported event is GPS detected or not.

All processing and analyses were carried out in R (v. 3.4.4) [73]. Specifically, we used the R packages plyr, dplyr, reshape, sp, dbscan, data.table, aspace, geosphere, and raster for data manipulation; ggplot2, maptools, knitr for graphs and visualizations; and corrplot, PerformanceAnalytics, ICC, rmcorr, psych, lme4 for the statistical analyses.

## 4. Results

### 4.1. Included Data

Reported TOH and AL events went through different validation processes as detailed in Section 3.3. Out of the initial 324 reported TOH events, 305 TOH events remain after exclusion of 18 events because of short duration and/or happening outside the study period (Table 2). 543 ALs were retained as valid reported ALs out of a total of 817. A total of 274 AL events were excluded because they met least one of the criteria detailed in Table 2. After exclusion of the invalid study days and participants (as described in Section 4.2), the valid reported number of events eventually retained for further analysis was 223 (minus 82) for TOH, and 437 (minus 106) for #ALs.

**Figure 6:**
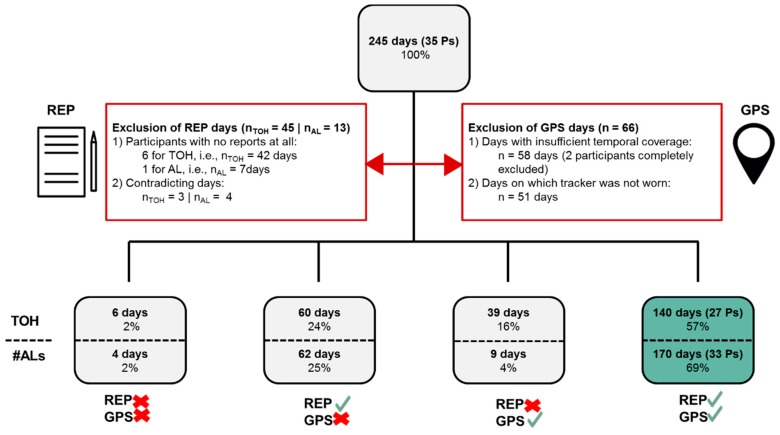
Exclusion of initial number of study days and participants (Ps) (top box) based on inclusion criteria for reported data (REP, left red box) and GPS data (right red box); and number of resulting days with no valid data at all, only valid reported data, only valid GPS data, and matching valid REP and GPS data (bottom boxes).

Based on the pre-filtered events, study days and participants were checked for validity based on the inclusion criteria detailed in Section 3.6 (Figure 6). Out of the original 35 participants (245 days), 27 participants (140 days) for TOH and 33 participants (170 days) for #ALs were included.

Participants had between 2 and 7 valid study days, with an average of valid days of 5.1 (standard deviation (SD) = 1.7) and 4.9 (SD = 1.6) for TOH and AL, respectively. Summary statistics for the daily GPS-based and reported mobility indicators shown in Table 3 as well as further analyses, are based on the participants/study days that fulfilled the inclusion criteria.

**Figure 7:**
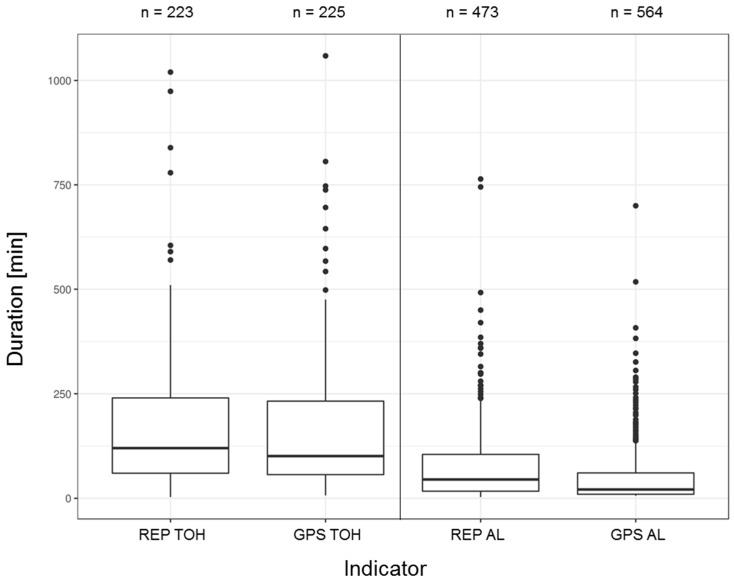
Number of events (n) over all valid study days and boxplots showing the distribution of event duration by mobility indicator and assessment methods.

Figure 7 shows the distribution of event duration for both TOH and AL events based on both assessment methods. As was to be expected, the median event duration is considerably longer for TOH (REP: 120.0 min vs. GPS: 100.9 min) than for AL (REP: 45.0 min vs. GPS: 21.1 min). The number of GPS events (especially for AL) tend to be higher than their reported counterparts. Median durations are considerably lower for GPS than for REP events.

### 4.2. Sensitivity Analysis

**Figure 8:**
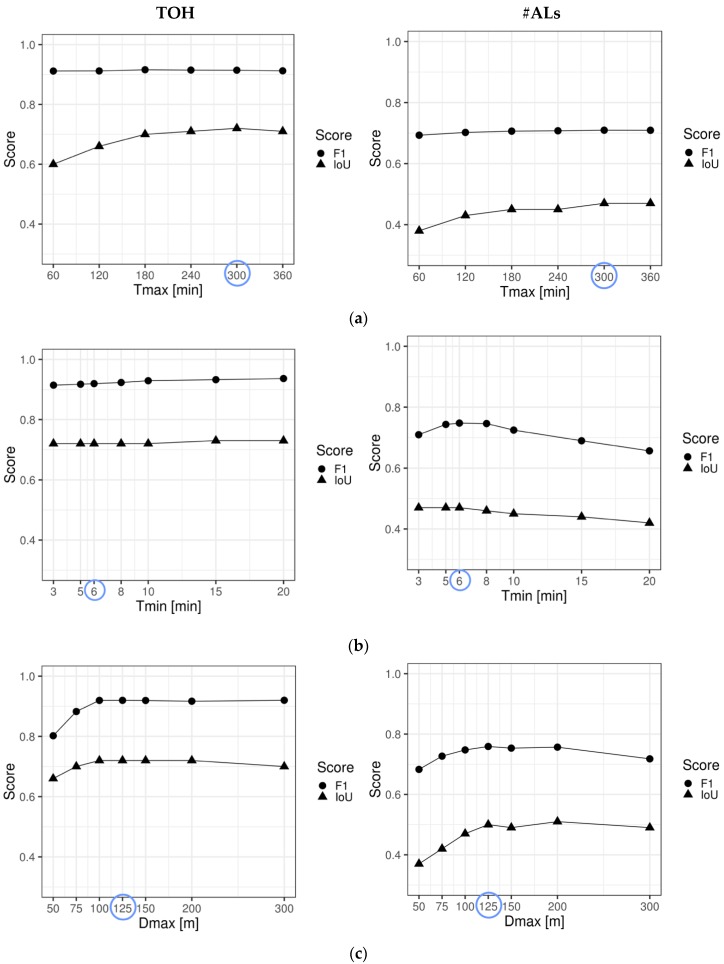
Results of stepwise sensitivity analysis of *Tmax*, *Tmin*, and *Dmax.* (**a**) Optimization of *Tmax* for initial settings (*Tmin* = 3 min, *Dmax* = 100 m). (**b**) Optimization of *Tmin* for initial *Dmax* = 100 m and optimized *Tmax* = 300 min, (**c**) Optimization of *Dmax* for optimized *Tmin* = 6 min and *Tmax* = 300 min.

The results of the stepwise sensitivity analysis of (1) *Tmax*, (2) *Tmin*, and (3) *Dmax* are displayed in Figure 8.

1. While the count-oriented agreement measure F1 barely reacts to varying *Tmax*, the duration-oriented IoU steadily increases, with an optimum at 300 min (i.e., 5 h) for both TOH and #ALs. For *Tmax* between 180 and 360 min (i.e., 3 and 6 h) indicator agreement appears to be stable.
2. While *Tmin* has no major impact on agreement between REP and GPS indicators, it does affect the indicator agreement for #ALs. F1 (the count-based measure more relevant for #ALs) seems to peak between 5 and 8 min (maximum at 6 min) while IoU starts dropping at 8 min.
3. For TOH, indicator agreement is increasing until *Dmax* = 100 m and then remains stable, only starting to slowly drop at 300 m. For #AL, the best indicator agreement is obtained for *Dmax* = 125 m. After 200 m, both measures of agreement start dropping.

As expected, both indicators, TOH and #ALs show similar fluctuations in indicator agreement to the variations of all three thresholds. Despite subtle differences in the reaction to threshold variation, we used the following same thresholds for both indicators in further analyses at which both indicators jointly reached a maximum: *Tmax* = 300 min, *Tmin* = 6 min, *Dmax* = 125 m. Moreover, these optimized thresholds have been confirmed by reversely varying *Tmin* and *Tmax* (see Figure A4 in Appendix D).

### 4.3. Agreement between Daily REP and GPS-Based Mobility Indicators

**Figure 9:**
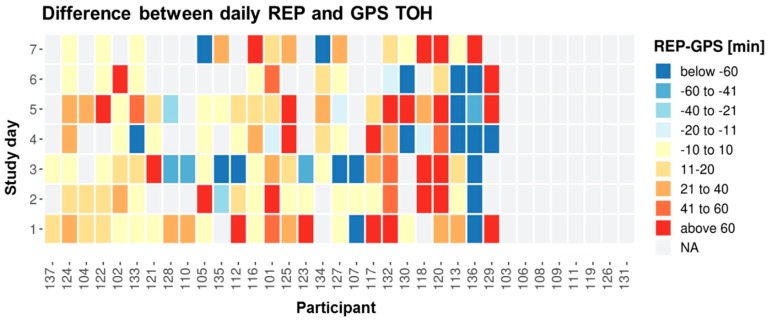
Daily difference between REP and GPS TOH by participant and study day: Yellow = agreement, blue shades = REP < GPS, red shades = REP > GPS, grey = invalid days, all grey = invalid participants (**#103, 106, 108, 109, 111, 119, 126, 131**). Participants are ordered according to increasing average absolute deviation per participant (ignoring NAs). Participants are represented by their unique user IDs.

**Figure 10:**
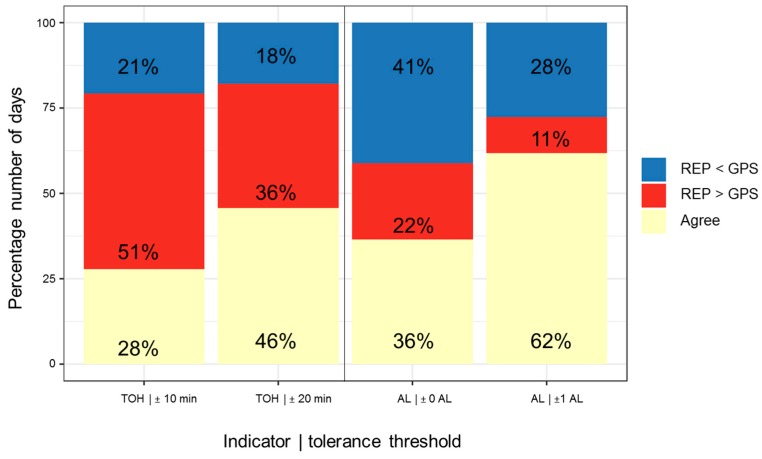
First and second bar from the left: Percentage number of TOH days with agreement between indicators, REP > GPS (red) and REP < GPS (blue) for deviation tolerance thresholds of 10 min and 20 min (n = 140 days). Third and fourth bar: same for #ALs, with deviation tolerance thresholds of 0 and 1 activity location (n = 170 days).

**Figure 11:**
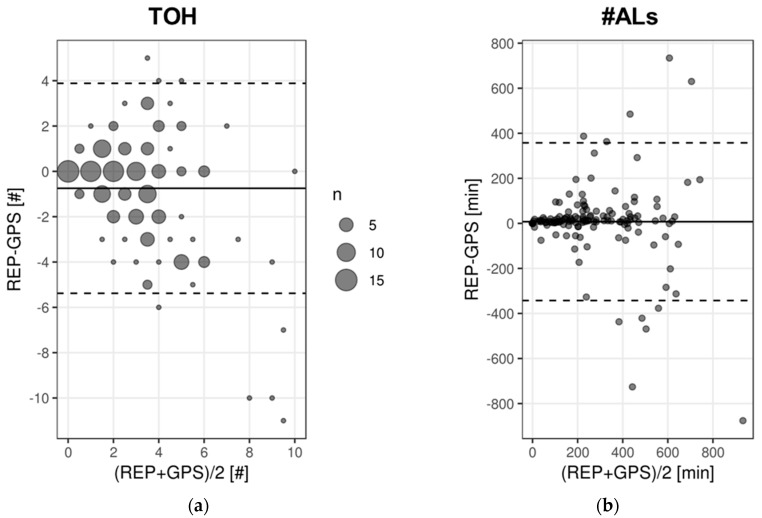
Bland Altman for multiple observations per participant [72] for TOH (n = 140 days) (**a**) and #ALs (n = 170 days) (**b**).

There is an average difference between daily REP TOH and GPS TOH of 7.5 min, with a large SD of 178.3 min (Table 4). Despite a lot of uncertainty given the large SD, the general trend goes toward REP durations to be longer than GPS durations. A relatively higher proportion of red shades to blue ones in Figure 9 also confirms the tendency for REP TOH to be higher than GPS TOH. Also, the comparison of the proportion of days on which REP was higher than GPS to the days on which the opposite was true, confirms this general trend toward REP TOH to be higher than GPS TOH (Figure 10). Accepting a time difference of 10 min between daily REP and GPS indicators as agreement, 51% of the study days are characterized by REP TOH being longer than GPS TOH, while only 21% are characterized by REP TOH being shorter than GPS TOH. For a 20-min-threshold, these percentages shrink to 36% and 18%, respectively. A day-by-participant analysis (Figure 9) and the between- and within-participant SD (Table 4) reveal that there is considerable between-participant and even higher within-participant variation in daily agreement. The average between-participant SD of 85.5 min is smaller than the average within-individual SD of 118.4 min for daily deviation between REP and GPS (Table 4). Based on Figure 9, approximately one-third of the participants tend toward high indicator agreement while about another third of the participants tend to have lower daily indicator agreement. Bland-Altman (BA) statistics averaging across participant-days reveal that REP TOH is on average 7.5 min longer than GPS TOH, however, with a wide range of agreement of [−342.6 min; 357.6 min] (Table 5 and Figure 11). ICC for TOH is substantial with 0.66 [95%-CI: 0.54; 0.76], and mean iCorr with 0.66 and correlation across all days with 0.81 are moderate and highly positive (Table 5).

**Figure 12:**
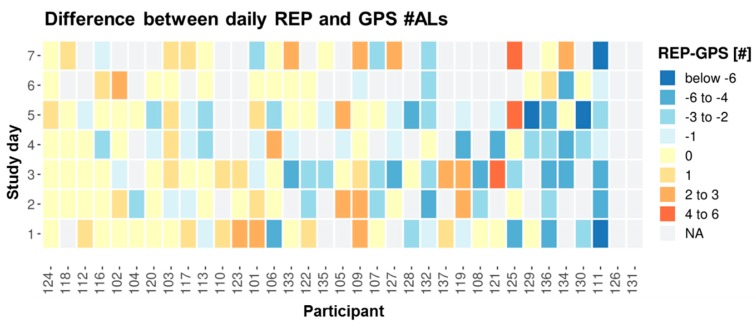
Daily difference between REP and GPS #ALs by participant and study day: Yellow = agreement, blue shades = REP < GPS, red shades = REP > GPS, grey = invalid days, all grey = invalid participants (**#126, 131**). Participants are ordered according to increasing average absolute deviation per participant (ignoring NAs). Participants are represented by their unique user IDs.

On average the daily REP #ALs is 0.7 below daily GPS #ALs (Table 4). This tendency of lower REP #ALs compared to GPS is also reflected in the participant-day plot (Figure 12); the plot shows a higher proportion of days colored in blue shades illustrating days with REP #ALs being smaller than their GPS-based counterparts. 41% or 28% of the days are characterized by REP #ALs being smaller than GPS, while only 22% or 11% of the days have REP exceeding GPS, depending on the deviation tolerance of disagreeing #ALs for a day at 0 or 1 location, respectively. In contrast to TOH, the #ALs plot represents less days with extreme disagreement by fewer days with extreme color. However, given the daily averages of 2.6 REP and 3.3 GPS #ALs (Table 3), a mismatch of a single AL is already critical. Clear differences in overall daily indicator agreement for #ALs between participants exist: approximately one-third of the participants with consistent agreement across days (i.e., about ±1 location), against another good third with high variation in daily indicator agreement against yet another small part of participants with reports falling below GPS on the majority of days. However, we also find here a significant portion of participants whose daily REP indicators at times agree, or at times are higher or lower than GPS, thus leading to high within-person variability in daily indicator agreement. Average SD for deviations between individuals with 1.5 is again slightly lower than the average within-participant SD of 1.8 (Table 4). Compared to TOH, there is a higher proportion of days with agreement between indicators of 36% or even of 62% for an accepted deviation of one location as agreement (Figure 10). Bland-Altman statistics suggest a mean difference of −0.7, with a wide LOA of [−5.4; 3.9] (Table 5). The BA plot also suggests a tendency toward clearly higher discrepancies between REP and GPS for days for which the average of GPS and REP indicators were high (Figure 11). ICC for #ALs was just below 0.50 and therefore moderate. Correlation (0.6) and average iCorr (0.53) were also moderate. However, the iCorrs are also characterized by a high SD of 0.5, pointing to large inter-individual differences in average daily indicator agreement.

### 4.4. Event-Centered Agreement between REP and GPS Mobility

**Figure 13:**
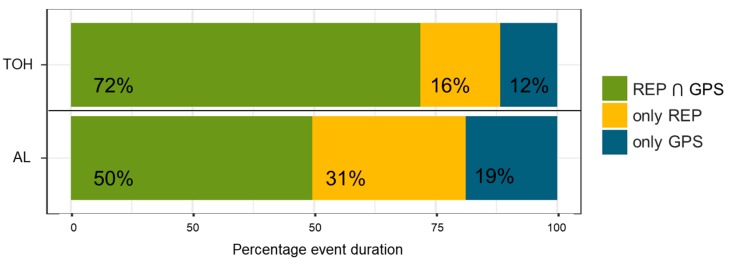
Percentage duration of matched, respectively, intersected (i.e., REP ⋂ GPS), REP only, and GPS only TOH/AL events (see Figure A3 Appendix B for illustration of computation). Percentages are computed for each participant based on all valid events of all valid days and then averaged over all participants.

For TOH, a count- (Table 6) and duration-based (Figure 13) matching comparison, respectively, of the indicators reveal high proportions of agreement. The majority (around 87%) of reported and GPS events have one temporally matching event detected from the corresponding opposite data source. Only a small number of events have multiple matching events or no matching event for both REP and GPS TOH. Also, the aggregated accuracy measures F1 (0.92) and IoU (0.72) reflect that indicators agree in clearly more cases, respectively for more duration, than they disagree.

In comparison to TOH for AL, considerably lower percentages of events have a matching event (59% for REP, and 68% for GPS). Equally high proportions of REP events remain undetected by GPS (24%) as GPS events remain without any matching REP events (25%). Moreover, especially for reported events, it seems to be a common issue that they temporally overlap with more than one GPS event (24%). F1 (0.76) and IoU (0.50) are considerably lower for AL than for TOH (Table 7).

### 4.5. Association between Reported Event Characteristics and Event Detection

Finally, in order to investigate whether certain reported activity locations have better chances of being detected, we investigated whether the event type or duration is associated with a reported event with at least one temporally matching GPS event (Table 8). Work-related and recreational activities seem to have high chances of being detected. However, only 5 work locations were reported, and the median event duration of work location is exceptionally long, which might increase the chances of being detected. Only transportation-related ALs have clearly lower detection rates. While most of the activity types have average overlap durations between 63% and 73%, lower average temporal coverage was found for transportation (40%), work (52%), and commercial activities (54%). Chances for an event to be detected seem not to be related to whether it was classified by participants as an indoor or outdoor location (Table 9). However, durations with matching overlapping GPS events tend to be higher for indoor (61%) events than for outdoor (49%) events. A separate correlation analysis showed little, however, significant correlation between reported event duration and whether an event was detected (Spearman’s rho of 0.29). A similar result was obtained for correlation between reported event duration and mean temporal overlap (Spearman’s rho of 0.27).

## 5. Discussion

As interest in the use of GPS-derived mobility indicators for applications in health and aging research is growing, more knowledge is needed about how to extract those features from GPS data. Likewise, GPS-based indicators need to be validated in comparison to self-reported data sources. In order to obtain more information on the validity of the two indicators TOH and #ALs, first, we demonstrated how the threshold choice for the TOH/AL event detection algorithm affects the indicator agreement and, second, examined in depth to which degree the daily REP and GPS-derived indicators compare.

### 5.1. Sensitivity Analysis: Which Thresholds Maximize Indicator Agreement?

Considering missing consensus in health and aging research regarding the temporal (*Tmax*, *Tmin*) and spatial (*Dmax*) thresholds used to compute mobility indicators, we evaluated how varying the individual thresholds affects the agreement between self-reported and GPS-derived mobility using a count- and a duration-oriented measure of agreement (i.e., F1 and IoU, respectively) (Figure 8). For *Tmax*, we found that temporal interpolation up to 5 h keeps improving indicator agreement, especially in terms of correspondence in temporal overlap reflected in IoU. The long interpolation intervals potentially address time spent indoors where GPS signals are frequently lost, and this would help identify longer periods of correspondence between REP and GPS. This is critical for duration-oriented mobility indicators such as TOH, but many applied studies currently use no or only short temporal interpolation of at most 1 h [2,9,19,45]. Thus, the result with *Tmax* ≤1 h may underestimate the GPS-based mobility indicators. Further, complex indicators such as time spent in traveling using different transport modes may require more sophisticated interpolation methods that allow filling in the missing GPS fixes.

For *Tmin*, highest indicator agreement between REP and GPS was found between 5 and 6 min. One explanation for the lower agreement for shorter time thresholds is that participants perceived events of shorter than 5–6 min as not sufficiently significant and therefore did not report them (respectively forgotten to be reported), despite the instruction of reporting events that last for 3 min or longer. We also found that lower thresholds tend to over-segment the GPS trajectory, resulting in many false positive stops. Such stops could be short insignificant stops (e.g., traffic light, change of transport mode) or slow walking that might have been mistakenly detected as stops [17]. This result slightly deviates from the findings of Cich et al. [17] and Hwang et al. [66] who found best indicator agreement between 3 and 4 min using stop detection algorithms comparable to the one by Montoliu et al. [38] used in this study.

Finally, sensitivity analysis for *Dmax* showed that highest indicator agreement is obtained somewhere between 100 and 200 m for both TOH and AL indicators. This is in line with other studies that tested the sensitivity in varying the spatial extent defining an activity location, finding best indicator agreement for 100 m [66], 100–125 m [17], and around 200 m [37]. The choice of optimal thresholds is generally a function of the positional accuracy of the GPS data (lower accuracies requiring higher *Dmax*), the GPS sampling frequency (lower frequency requiring higher *Dmax* and *Tmin*), the applied algorithm (sequential ones typically using lower *Dmax* than global density-based clustering [37,46]), participants’ behaviors (e.g., slower walking speeds requiring higher *Dmax*), and the application domain that conceptually define the spatial and temporal extent of a stop. Moreover, *Dmax* and *Tmin* interact with each other: higher *Dmax* is likely to induce higher *Tmin*, and vice versa. This implies that if the interest is in finding more coarse-grained activity regions, higher values of *Tmin* and *Dmax* would have to be chosen [38]. In general, lower *Tmax* and *Dmax* values lead to a higher number of correctly identified events, but conversely also to a higher number of over-segmented stops and falsely identified or meaningless stops, respectively.

### 5.2. Agreement between Self-Reported and GPS-Derived Time Out of Home

Based on optimal threshold constellation for our application, we found, on average, participant-reported TOH to exceed GPS-based TOH by 7.5 min (Table 4). Accepting a 20-min difference between the daily REP and GPS TOH, given an average REP and GPS TOH of 277.6 and 270.1 min (approximately 4.5 h; Table 3), respectively, 46% of the valid days have corresponding indicators (Figure 11). The average daily REP and GPS TOH are in the order of magnitude of other studies on older adults, with REP and GPS TOH between 3.5 and 4 h [11,14,74].

The average variability in daily indicator agreement was higher within participants (118.4 min) than between participants (85.5 min). This suggests that reporting accuracy is not necessarily conditioned by participants’ general reporting accuracy (Table 4). It seems more likely that daily indicator agreement is influenced by daily factors, including daily varying mobility patterns or more random reasons, including device functioning. Nonetheless, measures of agreement of REP and GPS on daily TOH show substantial ICCs and high positive correlation across valid study days (Table 5). Compared to daily #ALs, REP, and GPS agree more for daily TOH. It means that TOH has higher convergent validity and is more stable over different assessment methods than #ALs.

The event-centered analysis generally confirms this result. A count-based perspective reveals that most of the REP and GPS events (87% for each) have a matching event in the respective opposite data source (Table 6). From a duration-based perspective still a high percentage of 72% of durations of REP and GPS indicators do agree (Figure 13). This means that while there seems to be high agreement between REP and GPS that there was an event at all, REP and GPS indicators agree to a slightly lower degree upon exact durational overlap of the respective events. Reasons for deviations in duration can be attributed to both sides, i.e., participants not correctly recalling start/end times of events or incorrect GPS-based TOH event detection because of positional inaccuracies, combined with gaps in the GPS data. Especially, if there is missing GPS data at the beginning or end of an event, no interpolation can be carried out. For daily TOH assessments, slightly shifted REP and GPS events would be of minor importance, as long as they are of equal duration.

Our results appear to be in line with previous research [51] that generally finds reports to exceed GPS-based out-of-home activities, especially known for the case of travelling. Klous et al. [53] compared self-reported to GPS-derived time outdoors (i.e., outside of any building, in contrast to TOH in our study, which focuses on the time spent outside the home, irrespective of whether this takes place indoors or outdoors). They found the reported to considerably exceed the GPS-derived time (median self-report 4 times higher than median GPS duration), while the average deviations we found are of an acceptable extent.

### 5.3. Underestimation of Daily Number of Activity Locations in Self-Reports

In contrast to TOH, daily self-reported #ALs on average fall below their GPS-based counterparts by 0.7 locations (Table 4). In general, there is a clear trend for higher deviations between REP and GPS #ALs on days with higher averages of REP and GPS #ALs. The deviations between REP and GPS are substantial, considering the average number of daily locations visited (2.6 and 3.3 according to REP and GPS, respectively) (Table 3). Also, the wide LOA from the Bland Altman analyses and the moderate ICC (0.47) and correlation values (correlation over all participant days of 0.6 and average iCorr of 0.53) indicate lower agreement for #ALs than for TOH (Table 5, Figure 12). Nevertheless, the degree of agreement is comparable, yet still slightly higher than the one found by Wolf et al. [16], who found 0.4 correlation between the GPS- and diary-based number of visited locations aggregated over the entire study period of each participant.

At the event level, the number of REP and GPS events with a temporally matching event reveals that a considerable percentage of GPS-based events do not have a temporal match (25%) (Table 5). To some degree we expected that the average shorter event duration of AL in comparison to TOH might lead to lower chances of temporal overlap because of temporal offsets between events of both sources. However, the rather high degree of mismatch suggests other reasons, such as individuals not reporting some of the events because they perceived them insignificant, or forgetfulness in reporting, or errors in GPS-based detection (e.g., confusing slow walk segments as stops). On the other hand, there is also a considerable number of REP events with no matching GPS event (24%). Again, to some degree the combination of inaccuracies of reporting the temporal extent of events and the generally short event duration might have resulted in having less temporal overlap between REP and GPS events. However, to some degree also the thresholds chosen (minimum event duration of 6 min), noise in the GPS signal or gaps in the GPS signal and therefore undetected events, may contribute to the explanation. Finally, also the number of REP events with multiple matching GPS events is considerable (17%). This may be due to a combination of GPS gaps and positional inaccuracies resulting from indoor environments (e.g., jitter ≥150 m is possible in high-rise buildings [66]) that can lead to over-segmentation of the GPS trajectory. The general trend for GPS to measure a higher number of activity locations compared to self-reports seems to be in line with other research stating that distance-based algorithms tend to over-segment GPS trajectories [37,39]. The number of REP locations with a matching GPS event is 59%, which is comparable to the finding of Schmidt et al. [23] who found 60% of REP events having a match. Similarly, the precision (0.76) and recall (0.75) are in a range comparable to those identified elsewhere [41] (Table 7).

Finally, an important point concerns the high number of REP ALs that had to be excluded because of invalid self-reports. Out of the original 817 ALs, we excluded 189 (23%) ALs that we had identified as clearly non-stationary activities (Table 2). This shows that the conceptual definition of AL must have not been clear to all participants and that therefore participants might have also missed out on reporting ALs that were detected by GPS. Another reason could be that participants tried to diligently report all executed activities including erroneously non-stationary ones, inclined to report too many rather than too few. Overall, for AL we can conclude that convergent validity could only be found to a limited degree. This result, however, seems to be in line with what was reported in comparable research [16,23,32,41].

### 5.4. Similar Detection Rates for Indoor and Outdoor Activity Locations

An exploratory analysis of the different activity types performed in a location exhibits that work and recreational ALs (including sports-related activities or going out for drinks or food) have particularly high probabilities of having matching GPS events (Table 6). Above-average event durations, especially for work-related activities, arguably increase chances of having a temporally matching GPS event. In contrast, a clearly lower proportion of the transportation-related ALs have a temporally matching GPS event. The typically short nature of transportation-related activities against the minimum stop duration defined in our event detection algorithm (*Tmin* = 6 min) are part of the explanation why they tend to remain undetected or at least temporally offset. Interestingly, we did not find any significant difference in the detection rate of ALs that individuals had reported as indoor versus outdoor. The idea that outdoor ALs might be better detected because of lower chance of inaccurate and interrupted GPS signal might be outweighed by the fact that outdoor ALs (e.g., visiting a market, outdoor sports facility, forest) are less clearly defined in space and therefore harder to be detected by our algorithm. Finally, small but significant associations were shown between event duration and whether it was detected or not, a pattern that was also found by Neven et al. [32], comparing GPS to diary-based trip duration.

### 5.5. Incomplete Self-Reported and GPS Data

One of the limitations of GPS-based studies is their high attrition rate because of technical issues (data outage due to signal interruptions or limited battery) or participant compliance (not wearing devices) [75]. Such issues could partly be solved by less battery draining sampling regimes (e.g., generally lower sampling frequency or reduced sampling frequency in stationary mode) or by providing better instructions to participants. In the present study, 66 days (27%) had to be excluded because of the device reported as unworn for at least one TOH event on the respective day (n = 51 days) or because of not long enough registration periods (n = 58 days) (Figure 6). For self-reports, 45 (18%) and 13 (5%) of the self-reported days were excluded for TOH and AL, respectively. We excluded self-reported days of participants who did not report any event at all (this was especially the case for TOH) and for days which showed inconsistent reporting (e.g., no REP TOH on days with REP ALs). Moreover, as stated in Section 2, daily self-reporting is demanding for participants and many reasons might lead to incorrect reporting. A higher number of AL events (as discussed in Section 5.3) were excluded compared to TOH events; this suggests that conceptually more complex aspects of mobility (in our case, AL events) also lead to less reliable reporting. This may have contributed to the results obtained in our sample, and it might be especially problematic for self-reports from older adults with cognitive impairments. However, data loss and incorrect reporting up to now seem to be a problem inherent to daily-life assessments that so far must be accepted in exchange for a higher ecological validity and the possibility of repeated observation, allowing for the study of within-person relationships [26].

### 5.6. Limitations and Future Directions

Several issues can be improved in future research. First, in this study, we used only GPS data to extract sensor-based mobility information. Combining various data sources might improve the accuracy in detected mobility features. For instance, integration of GPS with indoor sensors such as in Jansen et al. [76] would complement GPS-based outdoor mobility assessments with indoor mobility assessments. Indoor assessments are highly worth including for mobility-constrained populations who restrict most of their movement to indoor or close-to-residence environments (e.g., nursing home). Beyond location sensors, wearable video equipment could help assess micro-scale mobility and environments [77]; the real-time video analysis linked to GPS can enable a momentary survey via a mobile phone to confirm participants’ on-going activity and location and lead to improvement in sensing fine-scale mobility in space and time with semantic information [77]. Moreover, follow-up face-to-face interviews with the participants—despite being costly—could be used to verify and complete GPS-based assessments or to obtain more detailed semantic information regarding an individual’s mobility patterns. Do and Gatica-Perez [44], for example, invited participants to annotate activity locations that were previously identified based on their GPS data.

Second, our study has shown considerable variability both between participants and within a participant regarding daily indicator agreement. Future research with more participants could investigate whether individuals’ average indicator agreement is related to participants characteristics including sociodemographic characteristics [78], cognitive functioning [32], mobility-relevant characteristics (e.g., having access to a car), or characteristics with a potential impact on GPS data quality (e.g., living in a dense urban neighborhood with high buildings versus a rural area). For the daily within-person fluctuations in reporting agreement, one might investigate whether particular travel patterns (movement speed, duration, transport modes) or daily GPS data quality (number of records) have an effect on daily indicator agreement.

Third, this study has focused on convergent validity, which is only one way of assessing a construct’s validity. Further aspects need to be explored regarding whether methods reliably assess the intended construct, including studies looking at content and nomological validity [50]. Moreover, although we have looked at indicator agreement from different perspectives, we did not consider spatial overlapping between self-reported and GPS-based methods, as was done in other studies [18,19]. Besides temporal accuracy, spatial accuracy of mobility indicators is crucial when the goal is to eventually combine GPS-based information with environmental data in order to assess individuals’ health relevant environmental exposures.

Last but not least, this study has focused on the population segment of older adults for whom such mobility indicators are often, and increasingly, applied. However, we suggest that similar analyses should be repeated in younger adults, to evaluate whether higher indicator agreement may be found in this population segment. This would thus speak for younger adults to report more reliable mobility information.

## 6. Conclusions

We have assessed the convergent validity for two daily mobility indicators commonly used in health and aging research: time out of home (TOH) and number of activity locations (#ALs). To this end, we compared two methods to assess these mobility indicators, diary-based self-reports (REP) and GPS tracking. Sensitivity analysis was conducted to establish how thresholds affect agreement between self-reported and GPS-based mobility. Our results discovered that the agreement is maximally improved with temporal interpolations of GPS gaps of up to 5 h, a minimum stop duration of 5–6 min, and a maximum extent of an activity location between 125 and 200 m. For TOH, a radius (*Dmax*) to delineate home between 100 and 200 m resulted in highest indicator agreement. We found high agreement between daily self-reported and GPS-derived TOH, with self-reports in tendency slightly exceeding GPS-based daily TOH on average. For #ALs, only moderate agreement between daily reported and GPS-based indicators was found compared to TOH. This difference is partly attributed to the more complex nature of #ALs that requires accurate detection of multiple ALs. Further, on average we found higher daily GPS-based #ALs than self-reported #ALs. Considerable within- and between-individual variability in daily indicator agreement call for further studies identifying potential causes for disagreement. While both self-reported and GPS-based measures of TOH seem to agree and therefore deliver relatively reliable assessments, more complex mobility-related indicators such as #ALs should be used with caution, independently of the data source, or assessment methods used. It is essential that for each mobility indicator used, its validity in assessing the construct of interest is fully understood, in order to subsequently find valid associations with other health and aging relevant outcomes.

## Figures and Tables

**Table 1:** Advantages and disadvantages of self-reported diaries vs. location sensors to infer individual’s daily mobility.

	Advantages	Disadvantages
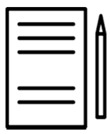	Semantic information (activity type, present persons) Subjective perspective	Errors/bias: retrospective/generalized/subjective responses, lack of care, recall bias, social desirability bias Active participant involvement (burden) Low spatio-temporal resolution
**Self-report (space-time diary)**		
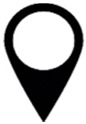	Assessments over long periods High spatio-temporal resolution Low participant burden Objective	Missing data because of technical issues (battery, satellite signal) Compliance issues (charging, wearing device) Lack of consensus on GPS data processing procedures
**Location sensor (GPS)**		

**Table 2:** Exclusion of self-reported TOH and AL events. Text in bold typeface indicates the main categories of retained events; plain text indicates categories of excluded events.

	TOH events	AL events
**Initial number of events**	**324**	**817**
Outside study period	18	50
Duration ≤3 min	1	16
Missing start/end time	–	14
Non-stationary/movement	–	189
At home	–	36
False reports	–	4
**Number of valid reported events**	**305**	**543**
Invalid study day	82	106
**Number of valid events of valid days**	**223**	**437**

**Table 3:** Summary statistics for daily mobility indicators over all valid participant days.

Variable	Method	Days	Mean	Median	SD	Min.	Max.
**Time out of home (TOH) (min)**	REP	140	277.6	245	207.1	0	1020
	GPS	140	270.1	216	224.6	0	1371
**Number of activity locations (#ALs)**	REP	170	2.6	2	1.8	0	10
	GPS	170	3.3	3	2.8	0	15

**Table 4:** Summary statistics for daily difference between self-reported and sensor-derived mobility indicators.

	Days	Mean	SD	Median	Min.	Max.	SD btw *	Mean SD within **
**TOH [min]**	140	7.5	178.3	11	−876	734	85.5	118.4
**#ALs [#]**	170	−0.7	2.4	0	−11	5	1.5	1.8

Note: GPS measures are subtracted from REP measures. Positive values therefore indicate REP > GPS while negative values indicate REP < GPS. * SD btw denotes the standard deviation between individuals’ average daily difference of the REP and GPS mobility indicators (n_TOH_ = 27 participants, n_AL_ = 33 participants). ** Mean SD within is the average of the within-individual standard deviation in their daily differences of the REP and GPS mobility indicators (n_TOH_ = 27 participants, n_AL_ = 33 participants).

**Table 5:** Measures of agreement for daily TOH and #ALs.

Measures of Agreement	TOH		#ALs	
Bland-Altman mean differences [95% LOA]	7.50	[−342.6; 357.6] (min)	−0.70	[−5.4; 3.9] (#)
ICC [95% CI]	0.66	[0.54; 0.76]	0.47	[0.34; 0.60]
Correlation Spearman	0.81	*	0.60	*
Mean (SD) iCorr Spearman	0.69	(0.41)	0.53	(0.50)

Note: Spearman correlation diagrams in Figure A5, Appendix E. * *p* < 0.01. LOA = limits of agreement; ICC = interrater correlation coefficient; SD = standard deviation. For TOH, n = 140 days respectively 27 participants, and for #ALs, n = 170 days respectively n = 33 participants, as outlined in Figure 6.

**Table 6:** Number of valid events with one match (1:1), multiple matches (1:n), no match (1:0).

Relation	REP TOH		GPS TOH		REP AL		GPS AL	
1:1	193	87%	196	87%	260	59%	383	68%
1:n	12	5%	11	5%	74	17%	42	7%
1:0	18	8%	18	8%	103	24%	139	25%
Total no. of events	223	100%	225	100%	437	100%	564	100%

**Table 7:** Measures of agreement on the level of events.

Event-Centered Measures of Agreement	TOH	#ALs
*Count-based*		
Precision	0.92	0.76
Recall	0.92	0.75
F1	0.92	0.76
*Duration-based*		
IoU	0.72	0.50

Note: The count-based measures were computed for the number of REP-/GPS-based events (n = 223/225 events for TOH and n = 437/564 for AL). IoU is first computed per individual over all events over all study days and then averaged over all participants (n = 27 participants for TOH and n = 33 participants for AL).

**Table 8:** Proportion of reported events that had a matching GPS event by reported event type (n = 437 events).

Activity Type	No. Events	No. Events with Match	Median Event Duration (min)	Mean Duration Overlap
Work	5	100%	360.0	52%
Recreation	55	93%	80.0	73%
Personal care	28	82%	45.0	64%
Social	32	81%	150.5	68%
Cultural/Religious/Education	41	78%	124.0	63%
Commercial	211	74%	30.0	54%
Transportation	41	56%	10.0	40%
Unassignable	24	–	–	–

**Table 9:** Proportion of reported events that had a matching GPS event distinguishing between indoor and outdoor ALs.

Type	No. Events	No. Events With Match	Median Event Duration (min)	Mean Duration Overlap
Outdoor	108	77%	32.5	49%
Indoor	320	76%	45.0	61%
Unassignable	9	–	–	–

**Figure A5:**
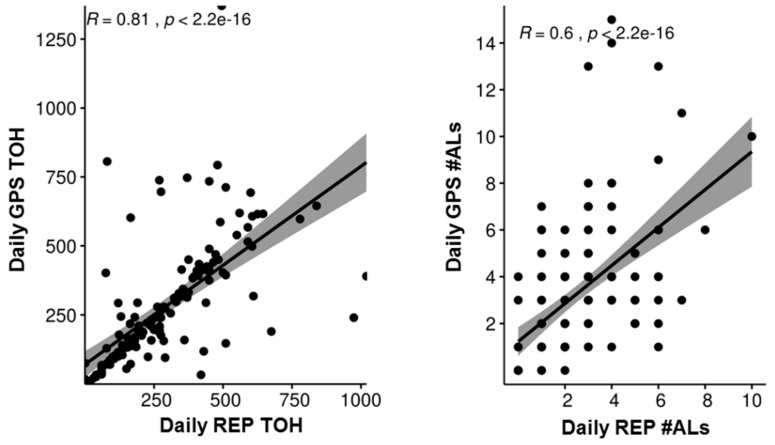
Correlations across all study days for TOH (**left**) and #ALs (**right**).

## Appendix A. F1 Adapted to Data with No Ground Truth [0,1]

This indicator evaluates how many events have a match at an event level. The number of events with a matching event are identified as TP1 or TP2 (green in Figure A1, blue in Figure A2). The number of events without matching event are identified as FN or FP (red in Figure A1, Figure A2). Based on these counts Precision, Recall, and F1 are computed in the following way:

- Precision = (*TP1*)/(*FP* + *TP1*) → how many of the self-reported events were identified by GPS-based events.
- Recall = (*TP2*)/(*TP2* + *FN*) → how many of the detected GPS-based events were actually self-reported.
- F1 = 2 × (Recall × Precision)/(Recall + Precision).

## Appendix B. Intersection Over Union (IoU) [0,1]

IoU evaluates the duration of temporal overlap (intersection; green in Figure A3) between self-reported (REP) and GPS-derived (GPS) (REP ⋂ GPS) events in relation to the duration with only REP (yellow in Figure A3) or GPS (blue in Figure A3) events.

- Intersection = duration of periods with temporal overlap between REP and GPS events (green periods in Figure A3)
- Union = duration of periods in which we have REP and/or GPS events (green, yellow, blue periods in Figure A3)
- IoU = Intersection/Union

## Appendix C. Measures of Agreement between Self-Reported and GPS-Based Mobility Indicators

**Table A1:** Description of measures of agreement between self-reported and GPS-derived mobility indicators.

Measures of Agreement		Description
Acronym	Full Name	
**BA**	Bland Altman difference	Bland–Altman statistic to compare methods with multiple observations per individual [72]. It plots the mean of daily self-reported (REP) and GPS-based mobility indicator against the difference between the two and computes the average difference as well as the 95% limits of agreement (LOA).
**ICC**	Interrater intraclass-correlation coefficient	We used the R package lme4 [79] to compute ICC in order to account for having multiple observations per participant. ICC divides true variance because of participants by total variance. Bootstrapping based on 1000 iterations was used to compute the 95% confidence intervals (CI) of the ICC.We used the following categories to interpret ICC values [80]: 0.00–0.20: slight 0.21–0.40: fair 0.41–0.60: moderate 0.61–0.80: substantial 0.80–1.00: almost perfect
**Corr**	Spearman correlation	Spearman correlation across all study daysWe used the following convention to interpret the strength of association [81]: |0.00|–|0.30|: little if any |0.31|–|0.50|: low |0.51|–|0.70|: moderate |0.71|–|0.90|: high |0.91|–|1.00|: very high
**iCorr**	Spearman individual correlation	Mean/SD of individual correlations [52] We used the same convention as for Spearman correlation to interpret association strength.
